# Clinical Utility of *GBA* Genotyping Prior to Deep Brain Stimulation: A Narrative Review

**DOI:** 10.3390/genes17010069

**Published:** 2026-01-06

**Authors:** Valentino Rački, Slaven Lasić, Filip Ðerke, Andrej Belančić, Matija Sošić

**Affiliations:** 1 Department of Neurology, Clinical Hospital Center Rijeka, 51000 Rijeka, Croatia; 2 Department of Neurology, Faculty of Medicine, University of Rijeka, 51000 Rijeka, Croatia; 3 Department of Neurology, University Hospital Dubrava, 10000 Zagreb, Croatia; 4 Department of Basic and Clinical Pharmacology and Toxicology, Faculty of Medicine, University of Rijeka, 51000 Rijeka, Croatia; 5 Special Hospital for Orthopedics and Rehabilitation “Martin Horvat”, 52210 Rovinj, Croatia

**Keywords:** GBA, deep brain stimulation, Parkinson’s disease, cognition, neuropsychiatric outcomes, genetic risk factors, patient selection, personalized medicine, pharmacoeconomics

## Abstract

**Background**: Variants in the *GBA* gene represent the most common genetic risk factor for Parkinson’s disease and are associated with a more aggressive disease course. Deep brain stimulation is an established therapy for advanced Parkinson’s disease, yet the influence of *GBA* status on postoperative outcomes remains incompletely defined. This review aims to summarize the clinical relevance of *GBA* genotyping prior to DBS and to evaluate its potential contribution to decision-making, risk stratification, and long-term management. **Methods**: A structured narrative review was conducted. The literature on sequencing methodology, variant interpretation, and postoperative outcomes in *GBA*-positive and *GBA*-negative patients was examined. Particular focus was placed on motor, cognitive, and neuropsychiatric outcomes, and on studies comparing trajectories across variant classes. **Results**: Across all study designs, patients with *GBA*-associated Parkinson’s disease demonstrated robust motor improvement after DBS, with outcomes comparable to those in non-carriers. Cognitive and neuropsychiatric decline occurred more rapidly in *GBA* carriers. Recent evidence indicates that cognitive and neuropsychiatric decline is influenced more by the genetic profile than the stimulation procedure. Variant severity appears to influence postoperative trajectories. Long-read sequencing improves detection of recombinant alleles and may refine genotype–phenotype associations. Genotyping provides additional value in counseling, expectation management, and postoperative planning. **Conclusions**: DBS remains an effective motor therapy for patients with *GBA*-associated Parkinson’s disease. Current findings indicate *GBA* genotyping should inform, and not limit, candidate selection. Integration of clinical, cognitive and genetic data supports more individualized management. Methodological advances in sequencing and the development of prediction models may further enhance personalized DBS planning.

## 1. Introduction

### 1.1. GBA Gene and Its Role in Parkinson’s Disease

Parkinson’s disease is a neurodegenerative disorder defined by bradykinesia, rigidity, and resting tremor. It is characterized by progressive degeneration of dopaminergic neurons in the substantia nigra and by intracellular aggregates of α-synuclein within Lewy bodies [1,2]. Both genetic and non-genetic factors contribute to disease risk. Variants of the *GBA* gene represent the most frequent and significant genetic susceptibility factor across all populations [3,4].

#### 1.1.1. Structure and Function of the GBA Gene

The *GBA* gene is located on chromosome 1q21 and contains 11 exons. It encodes the lysosomal enzyme glucocerebrosidase (GCase), which hydrolyses glucosylceramide and glucosylsphingosine into glucose and ceramide or sphingosine [2]. GCase is transported to the lysosome through its interaction with LIMP2, and its catalytic activity depends on Saposin C and negatively charged lipids. The mature protein consists of three structural domains that support folding and stability, with the catalytic site positioned within a TIM-barrel domain [2]. Multiple glycosylation sites contribute to proper trafficking and protein integrity.

#### 1.1.2. Variants of the GBA Gene and Their Impact on Disease Severity

A total of 300–500 *GBA* variants have been identified, including missense mutations, splice-site changes, small insertions or deletions, and complex recombination events with the adjacent pseudogene *GBAP* [2,3]. These variants differ markedly in their functional consequences. Severe variants, such as p.L444P, result in substantial loss of GCase activity and are associated with neuropathic forms of Gaucher disease. Mild variants, such as p.N370S, retain higher residual activity and cause non-neuronopathic disease. Risk variants, including p.E326K and p.T369M, do not lead to Gaucher disease but increase susceptibility to Parkinson’s disease [5]. Variant severity influences disease penetrance, age at onset, and clinical trajectory. Severe variants confer the highest risk for Parkinson’s disease and are associated with earlier onset and faster progression, while mild variants carry a lower but still significant effect. Overall, *GBA* carriers have an approximately five- to sixfold increased risk of Parkinson’s disease compared with non-carriers [3].

Furthermore, it should be noted that classification of GBA variants as severe, mild, or risk variants is not fully standardized across studies; the terminology used here reflects prevailing clinical conventions and is intended to facilitate integrative interpretation rather than imply uniform categorization across all publications.

#### 1.1.3. Pathophysiological Mechanisms and Clinical Symptoms

Several pathogenic pathways link *GBA* variants to Parkinson’s disease. Reduced GCase activity impairs lysosomal degradation and disrupts autophagy, which favors the accumulation and aggregation of α-synuclein [1,2]. Some variants produce misfolded GCase that is retained in the endoplasmic reticulum, which activates unfolded protein response pathways and contributes to cellular stress. Altered lipid homeostasis, mitochondrial dysfunction, and neuroinflammatory processes further enhance neuronal vulnerability [6]. These mechanisms are interrelated and converge on impaired α-synuclein processing.

Clinically, Parkinson’s disease associated with *GBA* variants shows a distinct trajectory. Carriers present with earlier symptom onset and a faster rate of motor and non-motor progression compared with non-carriers [5,7]. Cognitive impairment, psychiatric symptoms, autonomic dysfunction, REM sleep behavior disorder, and hyposmia occur more frequently. Severe variants are consistently linked with the most rapid decline and greatest cognitive burden. Neuropathological studies show a wider distribution of Lewy body pathology and colocalization of mutant GCase with α-synuclein in affected neurons, supporting the mechanistic link between impaired lysosomal function and α-synuclein aggregation [7]. Despite these group-level trends, clinical expression remains heterogeneous, and some carriers display milder disease courses.

### 1.2. Deep Brain Stimulation in Parkinson’s Disease

Deep brain stimulation (DBS) is an advanced treatment method that produces chronic electrical stimulation of deep brain structures and their pathways by way of stereotactically implanted electrodes. DBS involves reversible stimulation that mimics lesioning techniques of the basal ganglia, which were popular in the treatment of PD before the discovery of levodopa treatment. First reports of high-frequency stimulation of the VIM nucleus and STN nucleus by Benabid et al. [8] and Limousin et al. [9], which were positive, started a 30-year-old history of treating advanced PD patients with DBS. Today, STN-DBS is the most extensively studied intervention in treating advanced PD patients, with six RCTs against the best medical therapy, and exhibits the highest quality of evidence in recent European Academy of Neurology/Movement Disorder Society (European section) guidelines on therapies for the invasive treatment of PD [10].

Most DBS systems today use four contacts (two omnidirectional, two directional) on each bilaterally implanted electrode, connected with subcutaneous wires to an implantable pulse generator (IPG) surgically placed in the subcutaneous tissue underneath the collar bone. Current indications for initiating DBS treatment in advanced PD include patients with confirmed idiopathic PD who have refractory motor fluctuations and/or complications of chronic levodopa therapy (dyskinesia), have levodopa-unresponsive tremor, or are intolerant to dopaminergic medications [11,12]. Different timescales of DBS’s effect on Parkinsonian symptoms imply several different mechanisms. Tremor, rigidity, and bradykinesia respond to DBS in seconds to minutes, while axial symptoms require hours/days to weeks to fully respond [13]. It is these early responses to DBS treatment that led to the definition of the high-frequency informational lesional theory of the DBS effect. Beta band oscillatory activity (12–30 Hz) of sensory motor neural loops connecting the cortex, basal ganglia (STN, GPi), cerebellum, and thalamus leads to the promotion of tonic contraction, or motor resting state, a computational status quo in healthy individuals. With the initiation of movement, higher-frequency oscillations in the gamma range (30–100 Hz) and high-frequency oscillation ranges (100–500 Hz) dominate. In PD patients, pathologic beta band oscillatory activity predominates, even during movement, and entrains the whole network, leading to a computationally ineffective state resulting in bradykinesia and rigidity, while tremor is caused by the entrainment of pathological oscillatory activity of cerebello-thalamic fibers going through the VIM nucleus [14,15]. High-frequency STN or GPi stimulation disrupts the occurrence of pathological oscillatory activity, leading to better information processing in the thalamus and sensory motor cortex [14,15]. Studies with FDG-PET imaging proved that STN and GPi DBS change PD-related hypermetabolic patterns in the pons, globus pallidus, and thalamus, and hypometabolism in the premotor cortex, supplementary motor area, and parietal association areas [14,16,17]. This improvement in more global network activity leads to assumptions of neuroplasticity; however, modern hypotheses mostly reject this notion, as PD patients revert to the baseline state quickly after malfunction of the DBS system, especially after IPG battery failure [18]. This effect, on the other hand, more readily explains the slow-onset effect of DBS on axial symptoms. DBS is a symptomatic therapy with the main goal of improving quality of life. The success of DBS therapy rests on adequate patient selection, correct electrode placement, and regular follow-up with meticulous stimulation programming.

#### 1.2.1. Patient Selection

Most centers still select patients according to the CAPSIT–PD criteria published in 1999 [19]. The CAPSIT-PD criteria are potentially too strict and outdated, with recent data regarding genetic risk variants of PD and their influence on clinical progression placing additional strain on them [20]. Among the CAPSIT-PD criteria, presence of motor fluctuations and levodopa-induced improvement of motor symptoms are important criteria. Studies on patients with early motor fluctuations reported that DBS improvement was present only in patients with a worse quality of life (QoL) due to motor fluctuations, while patients without motor fluctuations experienced no improvement after DBS, even after 24 months [21,22]. Levodopa-induced improvement of motor symptoms informs both the patient and the clinician of what to expect from DBS treatment, as DBS improves only levodopa-responsive symptoms. Tremor, which is often L–DOPA-unresponsive, has a good DBS response [21,23]. L-DOPA-responsive axial symptoms, such as freezing of gait, will respond to DBS in the short term, but will continue to worsen due to disease progression. L-DOPA-unresponsive axial symptoms are a clear contraindication, as they represent advanced disease through sufficient neurodegeneration of non-dopaminergic neural networks [18,24,25]. Patients with cognitive impairment and older age have advanced neurodegeneration beyond dopaminergic neural networks, with lower QoL improvement with DBS, and also have higher bleeding risk, higher complication rates during surgery, and higher postoperative delirium [26,27]. Due to DBS’s heterogeneous effects on psychiatric issues (for example, it can lead to short-term improvements in depression, anxiety, impulse control disorders, and dopamine dysregulation syndromes, but can also induce apathy, stimulation-associated impulse control disorder, and unmet-expectation-related suicidality), it should be offered only to patients with a stable psychiatric state and reasonable expectations [28,29,30].

#### 1.2.2. Target Selection

Target nucleus selection is as important as good candidate selection. STN and GPi as targets produce satisfactory control of tremor, rigidity, and bradykinesia, while VIM is only effective in controlling tremor and is therefore used only in elderly tremor-dominant patients with stable disease and neuropsychiatric symptoms [11,31]. Significantly more studies have been published on STN-DBS compared to GPi-DBS [32]. The debate regarding whether STN or GPi is a superior target due to their similar motor effects is ongoing. Meta-analytical data from 2017 [33] indicate similar motor improvement in OFF and ON medication state according to the UPDRS II (activities of daily living) and UPDRS III (motor symptoms) questionnaires, with greater dopaminergic drug reduction associated with STN-DBS, while better control of depression and dyskinesia is possible with GPi-DBS. According to the EAN/MDS-ES guideline task force, both targets led to similar improvements in QoL measures (PDQ–39), activities of daily living (UPDRS II), and motor symptoms (UPDRS III), with similar complications of therapy, as well as side effects in RCT studies [10]. The possibility of greater dopaminergic drug reduction with STN-DBS (mean 50% reduction in the first year after surgery), and good long-term results, makes it a more favorable option in most centers [11], especially in younger patients with potential dopamine dysregulation syndrome. The downside of STN-DBS is its potential for neuropsychiatric side effects, dysarthria, and verbal fluency impairment. GPi-DBS is potentially more suitable and safer for older, frailer, mildly cognitively impaired patients with more severe dyskinesia, but it drains the IPG battery quicker, and long-term data are lacking compared to those for STN-DBS [11,33,34].

#### 1.2.3. Clinical Outcomes and Complications

Appendicular motor symptoms such as resting tremor, rigidity, and bradykinesia have a remarkable and sustainable response to DBS, including both STN-DBS and GPi-DBS [11,35]. The expected rate of improvement in the mean UPDRS III OFF state scores in the first 6 months to two years post-DBS surgery is 50%, with the best results for tremor (80% improvement) followed by rigidity and then bradykinesia (40–60% improvement). Dyskinesia is expected to improve by 40–88% following both STN-DBS and GPi-DBS. STN-DBS, due to the possibility of lowering the need for dopaminergic medication, and GPi-DBS, through direct stimulation, induced anti-dyskinetic effects [11,18,26,35]. Both STN-DBS and GPi-DBS improve time spent in the ON state by 50% (mean increase of 4 h), while time spent in the OFF state is improved by 60% (decrease of 3.5 h) [26]. Axial symptoms are notoriously difficult to control using DBS; however, initial improvement is expected to be up to 57% in the first year post-surgery, with expected progressive annual deterioration [26]. ON state improvement is more modest with DBS (15–25%), confirming the importance of the preoperative L-DOPA test for modeling predictions [26]. Long-term results have proven that STN-DBS leads to sustained improvements in motor symptoms and quality of life in a 5-year period [36], and even in a 15-year period [37]. DBS increases survival in PD patients (mean increase in survival of 7.6 months) while decreasing nursing home admissions, falls, and psychosis [18]. On the other hand, DBS’s effect on ON state motor symptoms generally plateaued or declined below the baseline 5 years after surgery, with axial symptoms such as freezing of gait, gait instability, and dysarthria becoming more prominent due to disease progression or stimulation side effects, leading to a novel “long-term DBS” phenotype [24,37]. DBS was primarily developed to control motor symptoms of advanced PD. According to the current literature, non-motor symptoms (NMSs) of PD, such as pain, hyperhidrosis, urinary urgency, constipation, sleep disorders, anxiety, and dopamine dysregulation syndrome, are generally improved by direct or indirect effects of DBS [38]. STN-DBS’s effect on impulse control disorders is ambiguous. New-onset apathy may develop in STN-DBS due to stimulation effects or reductions in dopaminergic medication. Cognitive impairment, such as declines in verbal fluency, attention, processing speed, learning, and working memory, is more pronounced after STN-DBS compared to GPi-DBS [30,39]. Weight gain after DBS was reported due to lowering of dyskinesia by the STN and GPi-DBS, but also by an increase in food motivation due to stimulation of the limbic part of the STN [28,38,39]. GPi-DBS had a greater tendency to improve depressive symptoms compared to STN-DBS; however, neither led to statistically a significant improvement in depressive symptoms according to meta-analytical data [40]. On that note, STN-DBS is associated with a small but not negligible increase in suicidality (attempted-suicide rate of 0.9–2%), usually associated with unmet expectations and prior depressive symptoms [26,39,41,42]. In addition to stimulation side effects and disease progression symptoms being poorly treated by DBS, hardware-related issues should be expected. Hardware-related complications such as infections, skin erosion, lead fracture, and electrode or IPG migration are reported in 4% to 20% of cases [39]. Battery end of life may lead to sudden DBS failure, which leads to sudden L-DOPA-resistant worsening of motor symptoms, constituting a movement disorder emergency treatable only by restarting DBS [39].

## 2. Methodological Concerns Regarding GBA Sequencing

Accurate sequencing of GBA presents methodological challenges due to the complex genomic architecture of the locus. The gene lies near its highly homologous pseudogene, GBAP, with which it shares approximately 96–98% sequence identity across the coding region. This homology affects both wet-lab and bioinformatic processes, resulting in artifacts, incomplete variant detection, and variability in reported frequencies of pathogenic alleles [2]. Given the clinical and research importance of GBA in Parkinson’s disease, as the most common genetic risk factor, the methodological limitations of available sequencing approaches must be understood to ensure accurate variant interpretation.

### 2.1. Sequencing Techniques

Short-read next-generation sequencing (sr-NGS) initially appeared to address the scale and cost limitations of Sanger sequencing; however, its application to *GBA* has revealed comparable difficulties. Standard bioinformatic pipelines frequently misalign reads originating from the pseudogene, particularly in the region spanning exons 8–11, where homology is highest. Such misalignment may lead to false-negative findings when reads derived from GBA1 align to *GBAP*, as well as false-positive variant calls when pseudogene reads are assigned to *GBA* [43,44]. Furthermore, widely used analysis pipelines such as the GATK best practices workflow have been shown to underperform in this region and may fail to identify complex recombinants entirely, as illustrated by the inability of standard WGS analysis to detect the RecNciI allele in a subset of samples [45,46].

Improved targeted pipelines such as the *Gauchian* algorithm have significantly advanced the reliability of short-read sequencing at this locus. By incorporating locus-specific alignment strategies and copy-number awareness, Gauchian has demonstrated superior accuracy compared with generic secondary analysis pipelines and reliably detects gene conversions, reciprocal recombinants, and other *GBAP*-derived sequence tracts [46]. Recent independent evaluation has demonstrated notable limitations. In a cohort with Sanger-validated genotypes, Gauchian missed several pathogenic variants and produced false wild-type or “no-call” outputs despite adequate coverage, while showing inconsistent performance depending on the reference genome build [47].

Long-read sequencing technologies, including those of Oxford Nanopore and Pacific Biosciences, provide the most robust resolution of the *GBA* locus to date. Their capacity to generate contiguous reads of the entire 7.6–8.9 kb gene enables precise discrimination between *GBA* and *GBAP*, facilitates phasing of variants on individual alleles, and allows direct detection of recombinant chromosomes and structural variation. Oxford Nanopore sequencing has been shown to reliably detect rare and common variants with high true-positive rates across multiple analytic pipelines, outperforming several short-read variant callers [48]. Similarly, PacBio HiFi sequencing demonstrates high accuracy with no false-positive or false-negative calls across large population cohorts, enabling comprehensive characterization of complex alleles relevant to Parkinson’s disease [45].

### 2.2. The Challenge of the GBAP Pseudegene

The methodological complexity of GBA sequencing is largely attributable to the presence of GBAP, a non-functional pseudogene located downstream of GBA. The pseudogene is nearly identical to *GBA* in both the exonic and intronic regions, particularly in the 3′ region of the gene, where the degree of identity reaches 98%. This extensive homology results not only in technical sequencing artifacts but also in biologically significant recombination events that further complicate variant interpretation. Both reciprocal and non-reciprocal recombination between *GBA* and *GBAP* have been described, producing a variety of structural variants, including gene conversions, gene–pseudogene fusions, and copy-number changes such as copy-number gains (CNGs) and losses (CNLs) [43,46]. These rearrangements may contain segments of pseudogene-derived sequences embedded within apparently intact *GBA* alleles, often presenting clinically as severe pathogenic variants. An example is the TRIP-SV allele, a reciprocal recombination event that produces a triplicated segment composed of alternating *GBAP* and *GBA* sequences. Although such alleles may not disrupt the functional *GBA* coding region, their presence can confound both PCR-based amplification and downstream alignment, and their true population frequency remains insufficiently characterized [49]. Recombinant alleles such as RecNciI, RecTL, and c.1263del55 + RecTL are frequently misclassified unless long-read sequencing or specialized analytic pipelines are employed, as their distinguishing features often consist of short sequence blocks identical to the pseudogene.

It is important to highlight that limitations of short-read sequencing approaches may directly influence reported genotype–phenotype associations in DBS outcome studies due to the incomplete detection of recombinant alleles, gene–pseudogene conversions, and copy-number variants. All these issues can lead to misclassification of variant severity or erroneous assignment of non-carrier status, particularly in cohorts relying on standard short-read pipelines [43,45,46]. Such misclassification may attenuate observed differences between GBA-positive and GBA-negative groups, obscure severity-dependent effects, or contribute to heterogeneity in cognitive and neuropsychiatric outcomes reported across studies [50,51,52]. We previously published a cohort study, where *GBA* variants were the most common genetic risk factor for PD, although we decided to perform confirmatory Sanger sequencing, which ultimately ruled out several variants that were initially called on short-read sequencing [53]. From a translational perspective, improved resolution provided by long-read sequencing enables more accurate stratification of genetic risk and may refine prognostic counseling and postoperative monitoring strategies in patients considered for DBS [3,48].

### 2.3. Interpretation and Classification of Variants

Interpretation of *GBA* variants is further complicated by the interplay between sequencing artifacts, structural complexity, and the heterogeneous clinical spectrum associated with different classes of mutations. Common missense variants such as p.N409S and p.L483P are typically straightforward to recognize, although numerous other variants located in the exon 8–11 region may represent pseudogene-derived sequence tracts rather than true point mutations. These “GBAP-like” variants arise from gene conversions or recombination events and cannot be reliably identified using approaches lacking haplotype resolution [43,46].

Structural variants add additional layers of interpretive complexity. Although copy-number gains may preserve an intact *GBA* coding region, they often coexist with pathogenic missense variants and may modify clinical expression. Conversely, copy-number losses that disrupt the coding sequence are typically pathogenic and associated with Gaucher disease or increased risk of Parkinson’s disease. Accurate classification of these alleles requires long-read sequencing or highly refined short-read pipelines, as conventional methods often misassign or fail to detect these rearrangements [54].

Variants of uncertain significance (VUSs) remain a substantial challenge. Population-based long-read studies have revealed numerous rare *GBA* variants whose clinical relevance is unknown. In a Norwegian cohort, long-read sequencing identified thirteen rare variants, with the majority classified as uncertain significance despite high sequencing accuracy [48]. Similarly, in a Luxembourg cohort, twenty-two variants were categorized as VUSs, highlighting the persistent gaps in functional and clinical annotation [45]. Their classification requires integration of population frequency data, structural modeling, enzyme activity correlations, segregation analyses, and, critically, high-fidelity sequencing to exclude recombinant architecture.

Consensus recommendations for genetic counseling emphasize that variant classification must be contextualized within the limitations of current sequencing technologies. Interpretation should incorporate information about variant severity, zygosity, and broader clinical phenotype, acknowledging that technical limitations may lead to misclassification or masking of complex genomic rearrangements [4].

## 3. Influence of GBA Gene Variants on DBS Outcome

### 3.1. Search of the Literature

A comprehensive search of the literature was conducted to identify studies evaluating the impact of *GBA* (glucocerebrosidase) gene variants on clinical outcomes following deep brain stimulation (DBS) in Parkinson’s disease. The electronic databases PubMed and Embase were queried without restrictions on publication date, with the final search performed in November 2025. The search strategy combined controlled vocabulary terms and free-text keywords related to genetic status and surgical intervention. The primary search terms included “*glucocerebrosidase*”, “*GBA*”, “*GBA1*”, “*Parkinson’s disease*”, “*deep brain stimulation*”, “*DBS*”, and “*subthalamic nucleus*”. Boolean operators were applied to refine the search (e.g., “*glucocerebrosidase or GBA*” *and* “*deep brain stimulation or DBS*”).

Titles and abstracts were screened for relevance, followed by full-text evaluation of eligible studies. The inclusion criteria encompassed original research articles, retrospective or prospective observational studies, randomized or non-randomized clinical studies, cohort analyses, case series, and case reports describing postoperative motor, cognitive, neuropsychiatric, or quality-of-life outcomes in GBA-positive and GBA-negative Parkinson’s disease cohorts undergoing DBS. Studies focusing exclusively on animal models, basic science, or non-DBS interventions were excluded.

Reference lists of included manuscripts and relevant reviews were manually screened to identify additional eligible publications. Data extraction was performed independently by multiple reviewers, with discrepancies resolved through consensus. The final dataset reflects the largest and up-to-date mapping of *GBA*-related DBS outcomes in Parkinson’s disease.

Overall, the available literature comprises 18 original publications (Table 1), including retrospective and prospective cohort studies, a meta-analysis, case series, and case reports, encompassing more than 450 patients with GBA-associated Parkinson’s disease treated with deep brain stimulation. Across the included studies, STN-DBS was the predominant stimulation target [51,52,55,56]. Only a minority of cohorts included patients treated with GPi-DBS or VIM-DBS, and formal stratification of outcomes by target was performed in a limited number of studies.

### 3.2. Motor Outcome

Across all included studies, patients with *GBA*-associated Parkinson’s disease consistently demonstrated significant motor improvement following DBS, particularly with STN-DBS. Large retrospective and multicenter cohorts reported marked postoperative reductions in motor fluctuations, dyskinesias, and MDS-UPDRS III scores in *GBA* carriers, with motor benefits comparable to those in non-carriers [52,55]. Additional retrospective studies confirmed similar UPDRS III outcomes postoperatively regardless of *GBA* status [51,57]. Likewise, multiple analyses observed robust motor improvement in both *GBA* and non-GBA groups during the first postoperative year [56] and sustained improvements at longer follow-up intervals [58,59].

Meta-analytic evidence further supports reliable motor benefit in *GBA* carriers, showing good motor and pharmacological outcomes despite underlying genetic vulnerability [50]. Cohort-level analyses revealed that motor outcomes remained positive even when cognition diverged between genetic subgroups [60]. Propensity-matched datasets demonstrated comparable long-term motor trajectories between *GBA* and non-*GBA* patients, although slight worsening of OFF-medication/OFF-stimulation scores at five years was observed in *GBA* carriers [61]. Similarly, long-term follow-up identified no major differences in motor benefit across genetic groups, including LRRK2 and idiopathic PD, though *GBA* carriers had a higher risk of non-motor complications [62].

Smaller cohorts comparing *GBA* with other genetic forms of PD reported somewhat reduced motor improvement in *GBA* patients compared with *LRRK2* or *PRKN/PINK1/DJ1* groups [63]. Case-series and case reports uniformly documented sustained postoperative motor benefit in *GBA* carriers, including those with severe or rare variants such as L444P and G325R [64,65,66,67,68,69].

Overall, motor outcomes across all evidence levels show that DBS provides strong and durable motor benefits in *GBA* mutation carriers, comparable to those in non-carriers.

### 3.3. Cognitive and Behavioral Outcomes

Cognitive and behavioral outcomes show greater variability and consistently reveal increased vulnerability in *GBA* carriers. Multiple cohort studies observed faster cognitive decline following DBS in patients with *GBA* mutations, particularly in global cognition and frontal–executive function domains [51,55,56]. Longitudinal multicenter studies confirmed that *GBA* carriers experience steeper MDRS decline and higher rates of dementia over time [55,58]. While the largest multicenter study demonstrated that DBS itself does not accelerate cognitive decline beyond the intrinsic progression of *GBA*-associated PD, cognitive trajectories remained significantly worse than in non-carriers [52].

Prospective and retrospective cohorts further showed early postoperative cognitive deterioration in *GBA* carriers, with significant declines in attention, memory, conceptualization, and executive functions, and demonstrated considerably faster annual MDRS decline in *GBA* carriers over five years [59,60]. Meta-analytic evidence confirmed significant cognitive deterioration in STN-DBS *GBA* carriers, especially in those with severe variants, while GPi-DBS showed a milder cognitive profile [50].

Behaviorally, *GBA* carriers exhibited higher risks of psychosis, hallucinations, depressive symptoms, and other neuropsychiatric complications. Hazard ratios for psychotic episodes and cognitive decline were more than doubled in one long-term cohort [62]. Earlier data demonstrated universal cognitive impairment and high rates of depression in *GBA* STN-DBS cases at long-term follow-up [64]. Case reports highlight dramatic postoperative neuropsychiatric deterioration in severe *GBA* variants, including acute psychosis and rapid dementia after DBS [65]. Conversely, some milder variants showed stable cognitive profiles over several years [66,67,69].

Target-specific analyses were reported inconsistently across studies. Where stratification was available, cognitive decline appeared more pronounced in STN-DBS cohorts, while GPi-DBS was associated with a comparatively milder cognitive profile; however, these observations are based on small sample sizes and should be interpreted with caution [50,58,59].

In summary, the literature consistently shows that cognitive decline and neuropsychiatric complications are more common and progress faster in *GBA*-associated PD after DBS, although DBS does not appear to be the primary driver of decline.

### 3.4. Disease Progression

The overall disease course in *GBA*-associated Parkinson’s disease is characterized by more rapid and aggressive progression, particularly in non-motor and cognitive domains. Multiple longitudinal DBS cohorts demonstrated accelerated decline in *GBA* carriers compared with non-carriers, independent of surgical intervention [55,56,58]. Importantly, long-term multicenter evidence indicates that the genetic background, and not DBS, primarily determines the trajectory of cognitive and neuropsychiatric deterioration [52].

Hazard ratios for dementia and psychosis were significantly higher in *GBA* carriers over extended follow-up periods [62]. Even in cases where motor responses remained robust, *GBA* carriers progressed more quickly toward cognitive impairment and behavioral complications. Across studies, GBA variant severity was generally classified using a pragmatic framework based on the Gaucher disease phenotype and residual glucocerebrosidase activity, with variants grouped as severe (e.g., p.L444P), mild (e.g., p.N370S), or risk variants (e.g., p.E326K, p.T369M) [3,5,7]. However, variant categorization was not uniform across studies. Some cohorts combined mild and severe variants, others excluded risk variants, and several did not stratify outcomes by variant class at all [50,51,52]. In addition, differences in sequencing methodology and limited resolution of recombinant alleles further contributed to heterogeneity in variant classification [45,46,48]. These inconsistencies should be considered when interpreting genotype–phenotype associations and may partially explain the variability in reported cognitive and neuropsychiatric outcomes

The evidence across all study types supports that *GBA*-associated PD is a distinct, more aggressive subtype, with faster progression of cognitive decline and neuropsychiatric burden, despite clear motor benefits from DBS.

## 4. Clinical Utility of GBA Genotyping

### 4.1. Genotype-Driven Decision-Making

Incorporating GBA genotyping into the DBS evaluation process adds an important layer to patient selection, because carriers display a clinical trajectory that differs from non-carriers in ways that have been described as relevant even after surgery. Across all studies summarized in this review, patients with GBA-associated Parkinson’s disease consistently experience sustained motor benefits after DBS. Multiple retrospective and multicenter cohorts demonstrate robust postoperative improvement in motor fluctuations, dyskinesias, and UPDRS III scores, with outcomes comparable to those in non-carriers [51,52,55,56].

The distinctive feature of GBA-associated Parkinson’s disease is not diminished motor efficacy, but rather, increased susceptibility to cognitive and neuropsychiatric decline over time. Several cohorts document significantly faster deterioration in global cognition and executive function domains in GBA carriers following STN-DBS [51,55,56,58,59], and meta-analytic findings confirm that this decline is especially pronounced in individuals carrying severe GBA variants, while GPi stimulation may be associated with a milder cognitive profile [50]. Although STN-DBS remains the most used target in *GBA* carriers, limited evidence suggests that GPi-DBS may represent a cognitively safer alternative in selected patients, underscoring the need for individualized target selection informed by genetic and neuropsychological risk [31,50,58,59]. Importantly, the most recent and largest multicenter longitudinal study showed that DBS does not accelerate cognitive decline beyond the natural progression determined by GBA status [52]. The risk appears to stem from the intrinsic biology of GBA-associated disease rather than from DBS itself.

Genotype-driven decision-making can help clinicians anticipate postoperative trajectories. Severe variants, such as L444P, may confer greater risk of rapid neuropsychiatric deterioration, as illustrated in case reports [65], while milder variants, including G325R, have been associated with stable cognitive outcomes over long-term follow-up [66,67,69]. Genotyping should therefore be used to refine counseling, set expectations, and tailor postoperative monitoring, rather than to exclude candidates who may still substantially benefit from surgery.

### 4.2. Goal of Therapy

As mentioned earlier in the manuscript, the main goal of DBS therapy is to reduce disabling motor complications and improve quality of life through sustained control of motor fluctuations, dyskinesias, and other levodopa-responsive symptoms. Current findings show that this goal is also attainable in GBA-associated disease, as motor improvement following DBS is consistently documented across all study types [52,55,56,60].

Where GBA carriers differ is in the longer-term motor gains and cognitive decline. Because cognitive and neuropsychiatric symptoms progress more rapidly in GBA-associated Parkinson’s disease, long-term quality-of-life trajectories may not mirror the excellent motor outcomes. Evidence demonstrates that cognitive progression is driven by the genotype itself rather than by DBS [52]. Considering this latest finding, preoperative discussions should emphasize that DBS improves motor function but does not modify the accelerated cognitive decline associated with GBA variants.

Clarifying the limits of therapy is essential. Unrealistic expectations have been linked to poorer psychiatric and emotional outcomes after DBS [28,29]. For GBA carriers, setting appropriate objectives helps ensure that patients and families understand that motor improvement is anticipated, but that cognitive vulnerability persists and requires careful long-term planning.

### 4.3. Multidisciplinary Approach

Evaluation of GBA-associated Parkinson’s disease patients for DBS requires a comprehensive multidisciplinary assessment that integrates clinical, cognitive, psychiatric and genetic information [67]. The general selection principles remain the same, including confirmed idiopathic Parkinson’s disease, levodopa responsiveness, and the presence of disabling motor fluctuations [11,12,19]. However, genotype-specific factors require certain attention.

Neuropsychological assessment is arguably more important in these patients, as GBA carriers, even when cognitive performance is similar to that in non-carriers at baseline, demonstrate higher rates of postoperative decline [55,56,59]. Early executive dysfunction, subtle memory deficits, or declines in attention may provide important prognostic information. Psychiatric evaluation is equally important. Long-term studies report increased risks of psychosis, hallucinations, and depressive symptoms in GBA carriers following DBS [62], and dramatic postoperative deterioration has been described in individuals carrying severe pathogenic variants [65]. Ensuring psychiatric stability and aligning expectations are therefore essential components of risk assessment.

Because outcomes vary substantially across different GBA variants, medical professionals must interpret genetic results within the broader clinical context. Some carriers experience early and severe decline, while others maintain stable cognition for years [66,67,69]. This heterogeneity highlights the need for individualized decision-making. When clinical, cognitive and genetic information are jointly evaluated, GBA genotyping becomes a useful tool for designing personalized postoperative follow-up pathways.

## 5. Pharmacoeconomic Aspect of GBA Genotyping and DBS

The pharmacoeconomic considerations discussed in this section are intended to be hypothesis-generating and perspective-based. They are derived from extrapolation of existing clinical and outcome data rather than from formal cost-effectiveness or health-economic modeling. DBS is generally cost-effective for advanced Parkinson’s disease when long-term motor benefits offset its substantial upfront costs. However, outcomes are heterogeneous, and carriers of common GBA variants face increased risk of cognitive and psychiatric decline [70,71,72]. These complications drive greater health-care utilization, earlier institutionalization, and caregiver burden, thereby undermining long-term cost-effectiveness.

In this context, incorporating a rapid, inexpensive screening method for prevalent GBA variants into the preoperative pathway could enhance allocative efficiency. The cost of targeted genotyping is negligible compared with surgical and device expenditures, yet the information it yields is substantial [73]. Identifying high-risk patients allows for avoidance of DBS in those with an unfavorable risk–benefit profile, or for tailored counseling, intensified follow-up, and anticipatory interventions where surgery is required. Even modest reductions in poor postoperative trajectories could meaningfully shift incremental cost-effectiveness ratios in a favorable direction.

To define the true health-economic value, prospective studies are required that integrate real-world genetic, clinical, and quality-of-life outcomes into robust cost–utility analyses. Such work should link longitudinal cohort data with decision-analytic models that account for direct medical costs, indirect costs related to caregiver time and productivity loss, quality-adjusted life years stratified by GBA status, and the prevalence of pathogenic variants across populations. Equity and ethical considerations must be explicitly addressed, particularly whether genotyping functions as an exclusion criterion or as a framework for tailored surveillance and shared decision-making. One should also bear in mind that currently existing outcome studies of GBA carriers are limited by small sample sizes, short follow-up, and heterogeneous endpoints, meaning that any preliminary economic modeling must be interpreted with caution until larger multicenter datasets are available.

Pharmacoeconomics, however, represents only one dimension of health technology assessment (HTA). For complex interventions and serious disease contexts, safety, efficacy, social and ethical implications, and quality-of-life outcomes are equally critical. Ethical and social factors, in particular, often weigh as heavily as cost in decision-making around treatments [74]. Patient-reported outcomes must therefore be incorporated into evaluations of DBS in GBA carriers, despite the methodological challenges, to ensure that assessments reflect lived experience and not merely health-system expenditures. This requires explicit integration into established HTA frameworks, such as NICE in the UK, ICER in the USA, or EUnetHTA in Europe, which increasingly emphasize patient-centered outcomes and ethical dimensions alongside economic modeling.

Evidence gaps include the long-term cognitive trajectories of *GBA* carriers after DBS and the extent to which early supportive interventions mitigate costs. Addressing these with multicenter prospective cohorts will be critical. While sequencing costs continue to fall, the assertion that omission of genotyping may become economically unjustifiable remains speculative and depends on assumptions about variant prevalence, test uptake, and the magnitude of avoided poor outcomes. Future evaluations should model these parameters explicitly before firm policy conclusions are drawn. Integrating low-cost *GBA* screening into DBS evaluation thus represents a promising strategy to improve value-based care by aligning an expensive technology with those most likely to benefit.

## 6. Future Directions and Conclusions

Future research should aim to define the variability in outcomes across the spectrum of GBA-associated Parkinson’s disease. Current evidence indicates that motor improvement after deep brain stimulation remains stable in most patients, including those with pathogenic variants, as demonstrated in several retrospective and multicenter cohorts [52,55,56,58,59]. Differences between studies suggesting additive cognitive effects of DBS and those supporting a genotype-driven model of cognitive decline can largely be reconciled by considering study design and methodological context. Early retrospective case–control studies often compared *GBA*-positive DBS cohorts with heterogeneous control groups and limited baseline cognitive characterization, which may have amplified apparent combined effects of genotype and stimulation [51,56]. In contrast, the largest and most methodologically robust multicenter longitudinal cohort demonstrated that cognitive trajectories in *GBA* carriers were primarily determined by genetic background rather than by DBS itself, with preserved motor benefit across genotypes [52]. Meta-analytic evidence supports this interpretation, indicating consistent cognitive vulnerability in GBA carriers after DBS, modulated by variant severity and target selection, but without definitive evidence of direct DBS-specific cognitive toxicity [50]. Collectively, the available evidence supports a model in which DBS remains an effective motor therapy in *GBA*-associated PD, while long-term cognitive outcomes seem to reflect the underlying genetic disease biology.

Longitudinal multicenter studies are needed to determine which clinical or neuropsychological markers predict postoperative cognitive deterioration, because current evidence shows substantial heterogeneity across variant classes [50,51]. Variant severity appears to influence postoperative trajectories, with severe mutations associated with accelerated decline [65], while milder variants may show long-term stability [66,69]. Emerging long-read sequencing technologies offer improved detection of complex alleles and recombinant structures and may allow more accurate genetic classification [45,46,48]

Overall, future prospective investigations should integrate clinical, genetic, and cognitive variables into structured prediction models to support individualized planning of treatment and follow-up. Future research should also examine whether early psychiatric intervention and structured cognitive monitoring improve outcomes, given the increased risk of psychosis and mood changes in GBA carriers after DBS [62,64]. Finally, health-economic studies are needed to determine whether routine preoperative genotyping improves decision-making and long-term care outcomes, as suggested in recent analyses of cost-effectiveness and health technology assessment [70].

Recent evidence has refined our understanding of how *GBA* variants influence outcomes after deep brain stimulation in Parkinson’s disease. The largest and most methodologically robust trial to date, by Avenali et al. [52], demonstrates that the risk of cognitive decline is driven by the underlying genetic biology rather than by DBS itself. Motor outcomes remain consistently favorable and comparable across genotypes, reinforcing that GBA-associated Parkinson’s disease retains responsiveness to neuromodulation.

These findings shift the clinical perspective from genotype-based exclusion toward genotype-informed optimization. *GBA* status emerges as an important factor, rather than a contraindication, for counseling, expectation management, and postoperative monitoring. The rapid methodological advances in *GBA* sequencing, including the improved resolution provided by long-read technologies, now enable more accurate detection of complex alleles and recombinant structures that influence clinical phenotype. As sequencing becomes more reliable and accessible, integrating *GBA* genotyping into preoperative assessment represents a realistic step toward more individualized DBS planning.

Finally, we can conclude that the most recent data support a stratified approach in which genetic, cognitive, and clinical markers can be jointly evaluated. DBS remains an effective therapy for GBA-associated Parkinson’s disease, but its long-term value is maximized when genetic risk is recognized early, communicated clearly, and managed through coordinated multidisciplinary care.

## Figures and Tables

**Table 1:** Overview of the clinical studies and case reports regarding GBA variants and DBS.

Reference	Study Type	Patient Number	Outcome	**Key Result/Follow-Up**
Avenali et al. [55]	Retrospective case–control study	GBA+ STN-DBS *n* = 73 STN-DBS *n* = 292	**Motor**: Marked motor improvement, significant reduction in fluctuations and dyskinesias. **Cognitive**: Faster worsening at three-year follow-up, overt dementia diagnosis in 11% non-GBA-PD vs. 25% GBA-PD at 5-year follow-up. **Cognitive test**: Mattis Dementia Rating Scale (MDRS).	**Follow-up**: 5 years. **Key result**: Patients with GBA mutations had a long-term benefit in terms of motor performance. Majority did not develop dementia.
Avenali et al. [52]	Multicenter retrospective controlled cohort study	GBA+ STN-DBS *n* = 109 STN-DBS *n* = 430 GBA+ *n* = 76	**Motor**: Marked and sustained motor improvement in both DBS groups; non-DBS GBA+ showed progressive worsening. Cognitive: Both GBA+ groups declined similarly. DBS did **not** accelerate cognitive decline compared to non-DBS. **Cognitive test**: MDRS.	**Follow-up**: Up to 5 years. **Key result**: Cognitive trajectory driven by genotype rather than DBS. Quality of life improved in DBS groups but worsened in non-DBS GBA+. STN vs. GPi and variant class showed no major differences.
Pal et al. [51]	Retrospective case–control study	GBA STN-DBS *n* = 58 GBA+ *n* = 73 STN-DBS *n* = 92 GBA- DBS- *n* = 128	**Motor**: Not longitudinally examined; similar postoperative UPDRS III regardless of GBA status. **Cognitive**: GBA+DBS+ subjects declined on average 2.02 points/yr more than GBA-DBS- subjects, 1.71 points/yr more than GBA+DBS- subjects, and 1.49 points/yr more than GBA-DBS+ subjects. **Cognitive test**: MDRS.	**Follow-up**: 3–5 years after surgery. **Key result**: Composite analysis suggests that the combined effects of GBA mutations and STN-DBS negatively impact cognition.
Pal et al. [57]	Retrospective cross-sectional study	GBA+ STN-DBS *n* = 11 STN-DBS *n* = 72	**Motor**: Similar UPDRS III scores to non-mutation carriers. **Cognitive**: Similar Mini Mental State Examination (MMSE) scores to non-mutation carriers. **Cognitive test**: MMSE.	**Follow-up**: Up to 2 years. **Key result**: Patients with GBA mutations had quicker DBS from disease onset than non-mutation carriers, with similar motor and cognitive outcomes.
Mangone et al. [56]	Retrospective case–control study	GBA+ STN-DBS *n* = 25 STN-DBS *n* = 143	**Motor**: Good motor outcome as measured by UPDRS III and UPDRS IV scores in both groups. **Cognitive**: More pronounced cognitive decline in GBA patients (−3.2 ± 5.1) compared to non-mutation carriers (−1.4 ± 4.4). **Cognitive test:** MDRS.	**Follow-up:** One year. **Key result:** GBA+ is associated with early cognitive decline after DBS. Cognitive decline in GBA mutation carriers was independent of age.
Lythe et al. [58]	Prospective case–control study	GBA+ STN-DBS *n* = 15 GBA+ GPi-DBS *n* = 2 STN-DBS *n* = 17	**Motor**: Trends of increased motor worsening in GBA patients compared to non-mutation carriers; these were not statistically significant. **Cognitive**: Statistically significant worsening with 70% prevalence in GBA patients compared to 19% in non-mutation carriers. **Cognitive test**: MDRS.	**Follow-up**: 7.5-year mean follow-up. **Key results**: These results associate GBA variants with more prevalent and more severe cognitive impairment and a greater burden of non-motor symptoms.
Angeli et al. [59]	Prospective case–control study	GBA+ STN-DBS *n* = 13 GBA+ GPi-DBS *n* = 2 GBA+ VIM-DBS *n* = 1 STN-DBS *n* = 65 GPi-DBS *n* = 2	**Motor**: Similar motor improvement between both groups (61.0 ± 18.3 percentage improvement in GBA vs. 68.5 ± 19.3 percentage improvement in non-mutation carriers). **Cognitive**: Longitudinal 5-year follow-up data were available for 35 individuals; all had undergone STN-DBS, and 6 had GBA mutations. The mean ± SD decline for patients with GBA mutations was 4.4 ± 7.3 points per year compared with 0.5 ± 0.9 points per year among non-mutation carriers. **Cognitive test:** MDRS.	**Follow-up**: 1-year follow-up for most patients. **Key result**: Similarly good motor outcomes, with worse cognitive outcomes in GBA patients. Subgroup of 35 patients (6 had GBA) showed significantly higher cognitive deterioration.
Asimakidou et al. [50]	Meta-analysis	GBA+ STN-DBS *n* = 118 GBA+ GPi-DBS *n* = 4 GBA+ VIM-DBS *n* = 1 STN-DBS *n* = 667	**Motor**: GBA carriers with STN-DBS have good motor and pharmacological outcomes. **Cognitive**: Statistically significant cognitive decline, more prominent if associated with severe mutations. GPi DBS patients showed minor cognitive decline. **Cognitive test**: MDRS and MMSE scores converted to MoCA using validated methods.	**Follow-up**: 2.4-year mean follow-up. **Key result**: GBA carriers undergoing STN-DBS had good motor and pharmacological outcomes, but they experienced the worst cognitive outcomes and the worst quality of life of the tested groups.
Fernández-Vidal et al. [60]	Retrospective cohort study	GBA+ STN-DBS *n* = 13 STN-DBS *n* = 96 total (non-GBA subdivided into fast and slow progressors)	Motor: Similar motor improvement across all groups; slow progressors had the greatest LEDD reduction. Cognitive: GBA+ patients showed greater decline in attention, conceptualization, and memory; fast-progressor non-GBA group showed early decline in executive functions. **Cognitive test**: MDRS, frontal Systems Behavior Scale (FrSBe).	**Follow-up**: Retrospective; 2004–2023 time period. **Key result**: GBA-PD patients experience more rapid cognitive decline despite good motor response. Baseline MDRS did not predict postoperative cognition.
Kamo et al. [61]	Retrospective cohort study with propensity score matching	GBA+ STN-DBS *n* = 54 (matched *n* = 50) STN-DBS *n* = 253 (matched *n* = 50)	Motor: Comparable improvement; GBA carriers showed slightly worsened OFF-med/OFF-stim scores at 5 years. Cognitive: No significant differences; MMSE remained stable in both groups. **Cognitive test**: MMSE.	**Follow-up**: Up to 5-year follow-up. **Key result**: No major motor or cognitive impact of GBA status on DBS outcomes. LEDD decreased similarly in both groups.
Anis et al. [62]	Retrospective case–control study	GBA+ STN-DBS *n* = 20 STN-DBS *n* = 64	Motor: No significant differences in long-term motor outcomes between genetic groups. Cognitive: GBA+ had increased risk of cognitive decline (HR 2.28). Psychiatric: GBA+ had increased risk of psychotic episodes (HR 2.76).	**Follow-up**: 7-year follow-up. **Key result**: GBA variants were associated with higher neuropsychiatric and cognitive burden, despite preserved motor benefit.
Wu et al. [63]	Retrospective cohort study	GBA+ STN-DBS *n* = 3 LRRK2 *n* = 9 Parkin/PINK1/DJ1 *n* = 6	Motor: All groups benefited, but GBA carriers had significantly smaller motor improvements (β −24.94 vs. LRRK2; β −23.61 vs. Parkin/PINK1/DJ1). Cognitive/QoL: Smaller PDQ-39 gains in GBA carriers. **Cognitive test**: MMSE and MoCA.	**Follow-up**: Up to 2-year follow-up. **Key result**: GBA carriers showed reduced motor and quality-of-life benefits compared with other genetic PD groups.
Weiss et al. [64]	Case series	GBA+ STN-DBS *n* = 3 STN-DBS *n* = 6	**Motor**: Marked motor improvement, significant reduction in fluctuations and dyskinesias. Increased incidence of axial symptoms in longer follow-up **Cognitive**: All GBA+ patients and 33% of controls developed cognitive impairment. All patients developed depression.	**Follow-up**: Up to 10-year follow-up. **Key result**: Long-term benefit in terms of motor symptoms, with later-onset axial symptomatology. All GBA+ patients developed cognitive impairment.
Palakuzhy et al. [65]	Case report	GBA+ GPi-DBS *n* = 1	Motor: Good motor response to GPi stimulation. Cognitive: Rapid severe decline—delirium, hallucinations, anxiety, and progression to dementia within months. **Cognitive test**: MoCA.	**Follow-up:** 7-year follow-up. **Key result**: Severe GBA mutation (L444P) associated with dramatic neuropsychiatric deterioration post-DBS, possibly influenced by multiple risk factors.
Racki et al. [66]	Case report	GBA+ STN-DBS *n* = 1	Motor: Marked and sustained motor improvement over three years, with reduction in motor fluctuations and dyskinesia. Cognitive: Stable neurocognitive profile; no major decline during follow-up. **Cognitive test**: MoCA.	**Follow-up**: 3-year follow-up. **Key result**: Successful DBS outcome in GBA-associated PD within Gaucher disease type 1.
Racki et al. [67]	Case report	GBA+ STN-DBS *n* = 1	Motor: Marked and sustained motor improvement over five years, with reduction in motor fluctuations and dyskinesia. Cognitive: Stable neurocognitive profile; no major decline during follow-up. **Cognitive test**: MoCA.	**Follow-up**: 5-year follow-up. **Key result**: Successful DBS outcome in GBA-associated PD within Gaucher disease type 1.
Neri et al. [68]	Case report	LRP10 + GBA variant carrier undergoing DBS *n* = 1	Motor: Good motor improvement post-DBS. Cognitive: Presence of Parkinson’s disease dementia; DBS yielded partial symptomatic benefit, but disease progression continued. **Cognitive test**: MMSE.	**Follow-up**: 4 years post-surgery. **Key result**: Highlights complexity of DBS decision-making in combined LRP10- and GBA-variant carriers and the need for careful cognitive monitoring.
Ledda et al. [69]	Case report	GBA G325R variant PD patient with STN-DBS *n* = 1	Motor: Sustained long-term motor benefit with reduction in off-periods and dyskinesias. Cognitive: Cognitive function largely preserved despite long disease duration. **Cognitive test**: MMSE.	**Follow-up**: 14-year follow-up. **Key result**: Good DBS outcome in this GBA G325R mutation carrier, supporting potential suitability for DBS.
**Group definitions**: GBA+ indicates PD patients carrying pathogenic or likely pathogenic GBA variants; GBA− indicates non-carriers. STN-DBS, GPi-DBS, and VIM-DBS denote DBS targeting the subthalamic nucleus, globus pallidus internus, or ventral intermediate nucleus, respectively. DBS− refers to non-DBS comparator cohorts. **Abbreviations**: DBS, deep brain stimulation; STN, subthalamic nucleus; GPi, globus pallidus internus; VIM, ventral intermediate nucleus; PD, Parkinson’s disease; GBA, glucocerebrosidase gene; UPDRS, Unified Parkinson’s Disease Rating Scale; LEDD, levodopa equivalent daily dose; MDRS, Mattis Dementia Rating Scale; MMSE, Mini Mental State Examination; MoCA, Montreal Cognitive Assessment; PDQ-39, Parkinson’s Disease Questionnaire-39; QoL, quality of life.				

## Data Availability

No new data were created or analyzed in this study.

## Abbreviations

The following abbreviations are used in this manuscript:

DBS	Deep brain stimulation
STN	Subthalamic nucleus
GPi	Globus pallidus internus
GBA	Glucocerebrosidase (gene)
GBAP	Glucocerebrosidase pseudogene
GCase	Glucocerebrosidase (enzyme)
NGS	Next-generation sequencing
sr-NGS	Short-read next-generation sequencing
WGS	Whole-genome sequencing
PET	Positron emission tomography
UPDRS	Unified Parkinson’s Disease Rating Scale
MDS-UPDRS	Movement Disorder Society Unified Parkinson’s Disease Rating Scale
LEDD	Levodopa equivalent daily dose
QoL	Quality of life
NMS	Non-motor symptom
VUS	Variant of uncertain significance
CAPSIT-PD	Core Assessment Program for Surgical Interventional Therapies in Parkinson’s Disease
RCT	Randomized controlled trial
IPG	Implantable pulse generator
PD	Parkinson’s disease
PDQ-39	Parkinson’s Disease Questionnaire
MMSE	Mini Mental State Examination
MDRS	Mattis Dementia Rating Scale
HTA	Health technology assessment
